# Association between dexamethasone treatment and the host response in COVID-19 patients admitted to the general ward

**DOI:** 10.1186/s12931-022-02060-3

**Published:** 2022-06-03

**Authors:** Justin de Brabander, Erik H. A. Michels, Christine C. A. van Linge, Osoul Chouchane, Renée A. Douma, Tom D. Y. Reijnders, Alex R. Schuurman, Tjitske S. R. van Engelen, Amsterdam UMC COVID-19 biobank study group, Michiel A. van Agtmael, Anne Geke Algera, Brent Appelman, Frank E. H. P. van Baarle, Diane J. C. Bax, Martijn Beudel, Harm Jan Bogaard, Marije Bomers, Peter I. Bonta, Lieuwe D. J. Bos, Michela Botta, Justin de Brabander, Godelieve J. de Bree, Sanne de Bruin, Marianne Bugiani, Esther B. Bulle, Osoul Chouchane, Alex P. M. Cloherty, David T. P. Buis, Maurits C. F. J. de Rotte, Mirjam Dijkstra, Dave A. Dongelmans, Romein W. G. Dujardin, Paul E. Elbers, Lucas M. Fleuren, Suzanne E. Geerlings, Theo B. H. Geijtenbeek, Armand R. J. Girbes, Bram Goorhuis, Martin P. Grobusch, Florianne M. J. Hafkamp, Laura A. Hagens, Jörg Hamann, Vanessa C. Harris, Robert Hemke, Sabine M. Hermans, Leo M. A. Heunks, Markus W. Hollmann, Janneke Horn, Joppe W. Hovius, Menno D. de Jong, Rutger Koning, Endry H. T. Lim, Niels van Mourik, Jeannine Nellen, Esther J. Nossent, Frederique Paulus, Edgar Peters, Dan A. I. Piña-Fuentes, Tom van der Poll, Bennedikt Preckel, Jan M. Prins, Jorinde Raasveld, Tom D. Y. Reijnders, Michiel Schinkel, Femke A. P. Schrauwen, Marcus J. Schultz, Alex R. Schuurman, Jaap Schuurmans, Kim Sigalof, Marleen A. Slim, Patrick Smeele, Marry R. Smit, Cornelis S. Stijnis, Charlotte E. Teunissen, Patrick Thoral, Anissa M. Tsonas, Pieter R. Tuinman, Marc van der Valk, Denise P. Veelo, Carolien Volleman, Heder de Vries, Lonneke A. Vught, Michèle van Vugt, Dorien Wouters, Koos Zwinderman, Matthijs C. Brouwer, W. Joost Wiersinga, Alexander P. J. Vlaar, Diederik van de Beek, W. Joost Wiersinga, Tom van der Poll

**Affiliations:** 1 Amsterdam UMC location University of Amsterdam, Center for Experimental and Molecular Medicine (CEMM), Meibergdreef 9, Amsterdam, The Netherlands; 2 Department of Internal Medicine, Flevo Hospital, Almere, The Netherlands

## Abstract

Dexamethasone improves clinical outcomes in COVID-19 patients requiring supplementary oxygen. We investigated possible mechanisms of action by comparing sixteen plasma host response biomarkers in general ward patients before and after implementation of dexamethasone as standard of care. 48 patients without and 126 patients with dexamethasone treatment were sampled within 48 h of admission. Endothelial cell and coagulation activation biomarkers were comparable. Dexamethasone treatment was associated with lower plasma interleukin (IL)-6 and IL-1 receptor antagonist levels, whilst other inflammation parameters were not affected. These data argue against modification of vascular-procoagulant responses as an early mechanism of action of dexamethasone in COVID-19.

**Supplementary Information:**

The online version contains supplementary material available at 10.1186/s12931-022-02060-3.

## Introduction

Dexamethasone decreases the risk of progression to invasive mechanical ventilation and mortality in hospitalized patients with coronavirus disease 2019 (COVID-19) requiring supplementary oxygen [1, 2]. For this reason, dexamethasone became standard of care in the Netherlands for hospitalized COVID-19 patients requiring oxygen in August 2020. Data on the mechanism of action underlying the beneficial effect of dexamethasone in COVID-19 is limited.

COVID-19 is associated with endothelial dysfunction and coagulation activation, accompanied by hyperinflammation [3]. In sepsis, corticosteroids inhibit inflammation and endothelial cell activation [4]. However, systemic use of glucocorticoids has been linked to venous thromboembolism, particularly pulmonary embolism [5]. Here, we sought to evaluate the early effect of dexamethasone on endothelial, coagulation and inflammatory responses after hospitalization for COVID-19 by comparing biomarker levels in patients admitted in the era before and after implementation of dexamethasone as standard therapy.

## Methods

Patient enrolment was done in one secondary and two tertiary hospitals from March 2020 to May 2020 (first wave of the Dutch COVID-19 outbreak, no dexamethasone) and from October 2020 to March 2021 (second wave, dexamethasone 6 mg daily for up to 10 days). Samples were included from the ELDER-BIOME study (NCT02928367) and the Amsterdam UMC COVID-19 biobank. Patients were eligible if they were admitted to a general ward with COVID-19 (confirmed by SARS-CoV-2 PCR), required oxygen support and had provided written informed consent. Exclusion criteria were readmission, transfer from another hospital, participation in an intervention trial and chronic steroid use. EDTA blood was obtained within 48 h of admission, and if possible 3–4 days after admission. Sixteen biomarkers were measured by Luminex multiplex assay (R&D Systems Inc., Minneapolis, United States) and stratified according to three pathophysiological domains: “endothelial cell activation and function”, “coagulation activation” and “systemic inflammation”. Analyses were performed using R statistical software (version 4.0.2). Biomarker data were log-transformed. Normally distributed data were analysed by Student’s t-test, nonparametric continuous data by Mann–Whitney U test, and categorical data by Fisher exact test. To correct for possible confounding factors, a multiple linear regression analysis was conducted using age, gender, length of symptoms, the time between admission and sampling, comorbidities (cardiovascular, pulmonary, diabetes, malignancy, chronic kidney disease, immune suppression), and disease severity scores (4C Mortality, CURB and MEWS score) as covariates. All significance testing of biomarkers was multiple testing corrected using the Benjamini-Hochberg (BH) method.

## Results

Of the 174 patients, 48 patients were enrolled during the first wave (not treated with dexamethasone) and 126 during the second wave (treated with dexamethasone before sampling; Table 1). The median time between the first dose of dexamethasone and sampling was 19 h [interquartile range (IQR): 15–35]. The time between hospital admission and study sampling did not differ between groups (median [IQR] of 1 [1, 2] day). Age, body mass index and chronic comorbidities were similar between groups, whilst the proportion of females was higher in the first wave. Baseline vital signs, clinical and radiology severity scores did not differ between groups. None of the patients was vaccinated, and none received anti-IL-6 antibody treatment. Routine laboratory measurements at baseline showed no differences, except for higher platelet counts in wave 2. Outcome parameters in terms of pulmonary embolism, intensive care unit admission, hospital length of stay and mortality were comparable between groups.

**Table 1:** Patient characteristics

	Wave 1 (no dexamethasone)	Wave 2 (dexamethasone)	p-value
	n = 48	n = 126	
Demographics			
Age (years)	60 [51–69]	63 [51–72]	0.59
Body mass index	28.7 [26.8–32.5]	28.6 [25.8–31.6]	0.52
Female gender	25 (52.1)	42 (33.3)	0.04
Symptoms prior to admission (days)	9 [7–12]	8 [7–10]	0.38
Days between admission and sampling	1 [1–2]	1 [1–2]	0.26
Comorbidities			
Cardiovascular	21 (43.8)	55 (43.7)	> 0.99
Pulmonary	9 (18.8)	29 (23.0)	0.69
Diabetes mellitus	7 (14.6)	26 (20.6)	0.49
Malignancy	2 (4.2)	7 (5.6)	> 0.99
Chronic kidney disease	1 (2.1)	7 (5.6)	0.57
Immune suppression^a^	0 (0.0)	3 (2.4)	0.67
Vital signs on admission			
Heart rate (beats/min)	92 [84–105]	91 [80–100]	0.16
Systolic blood pressure (mmHg)	128 [120–140]	134 [123–148]	0.09
Diastolic blood pressure (mmHg)	80 [76–88]	80 [72–88]	0.98
Respiratory rate (/min)	24 [21–30]	24 [20–28]	0.44
Temperature (°C)	37.5 [36.7–38.1]	38.0 [37.3–38.6]	0.01
Laboratory values on admission			
C-reactive protein (mg/L)	96 [48–147]	90 [61–141]	0.66
White blood cells (× 10^9^/L)	6.1 [5.3–8.4]	6.1 [4.8–7.8]	0.39
Neutrophils (× 10^9^/L)	4.86 [3.74–6.59]	4.80 [3.30–6.26]	0.46
Lymphocytes (× 10^9^/L)	0.92 [0.74–1.31]	0.90 [0.60–1.15]	0.38
Neutrophil-to-lymphocyte ratio	5.43 [3.52–8.32]	5.18 [3.21–8.00]	0.70
Platelets (× 10^9^/L)	244 [190–322]	212 [154–248]	< 0.01
LDH (U/L)	359 [277–432]	336 [286–420]	0.92
Blood urea nitrogen (mmol/L)	4.65 [3.70–6.28]	5.40 [4.35–7.20]	0.12
Disease severity on admission			
4C Mortality Score^b^	9 [7–11]	10 [7–12]	0.15
CURB-65 score^c^	1 [0–1]	1 [0–2]	0.45
MEWS	3 [2–4]	3 [2–4]	0.25
qSOFA score	1 [0–1]	1 [0–1]	0.47
CT severity score^d^	11 [9–15]	11 [10–15]	0.73
Treatment prior to sampling			
Dexamethasone	0 (0.0)	126 (100)	< 0.01
Prophylactic anticoagulation^e^	32 (66.7)	88 (71.0)	0.72
Therapeutic anticoagulation^f^	7 (14.6)	10 (7.9)	0.30
Remdesivir	1 (2.1)	3 (2.4)	> 0.99
Outcomes			
Pulmonary embolism^g^	5 (10.4)	7 (5.6)	0.43
ICU admission	5 (10.6)	19 (15.3)	0.59
Hospital length of stay (days)	5 [3–9]	6 [3–9]	0.86
30-day mortality	7 (14.6)	13 (10.7)	0.67

Data are shown as n (%) or median [interquartile range]

*ICU* intensive care unit, *qSOFA* quick sequential organ failure assessment, *MEWS* modified early warning score

^a^Defined as chronic immune suppression due to asplenia, HIV, bone marrow or solid organ transplant

^b^Validated COVID-19 severity score [11]

^c^Clinical score used in community-acquired pneumonia, using confusion, blood urea nitrogen, respiratory rate, blood pressure and age

^d^Radiological scoring system to estimate the pulmonary involvement by COVID-19, with a maximum of 25

^e^Nadroparin 2850 IE or 5700 IE once daily, according to body weight

^f^Nadroparin 9500 IE twice daily, direct oral anticoagulant, continuous heparin infusion or vitamin K antagonist

^g^Diagnosed by CT pulmonary angiography within 28 days of admission

**Fig. 1:**
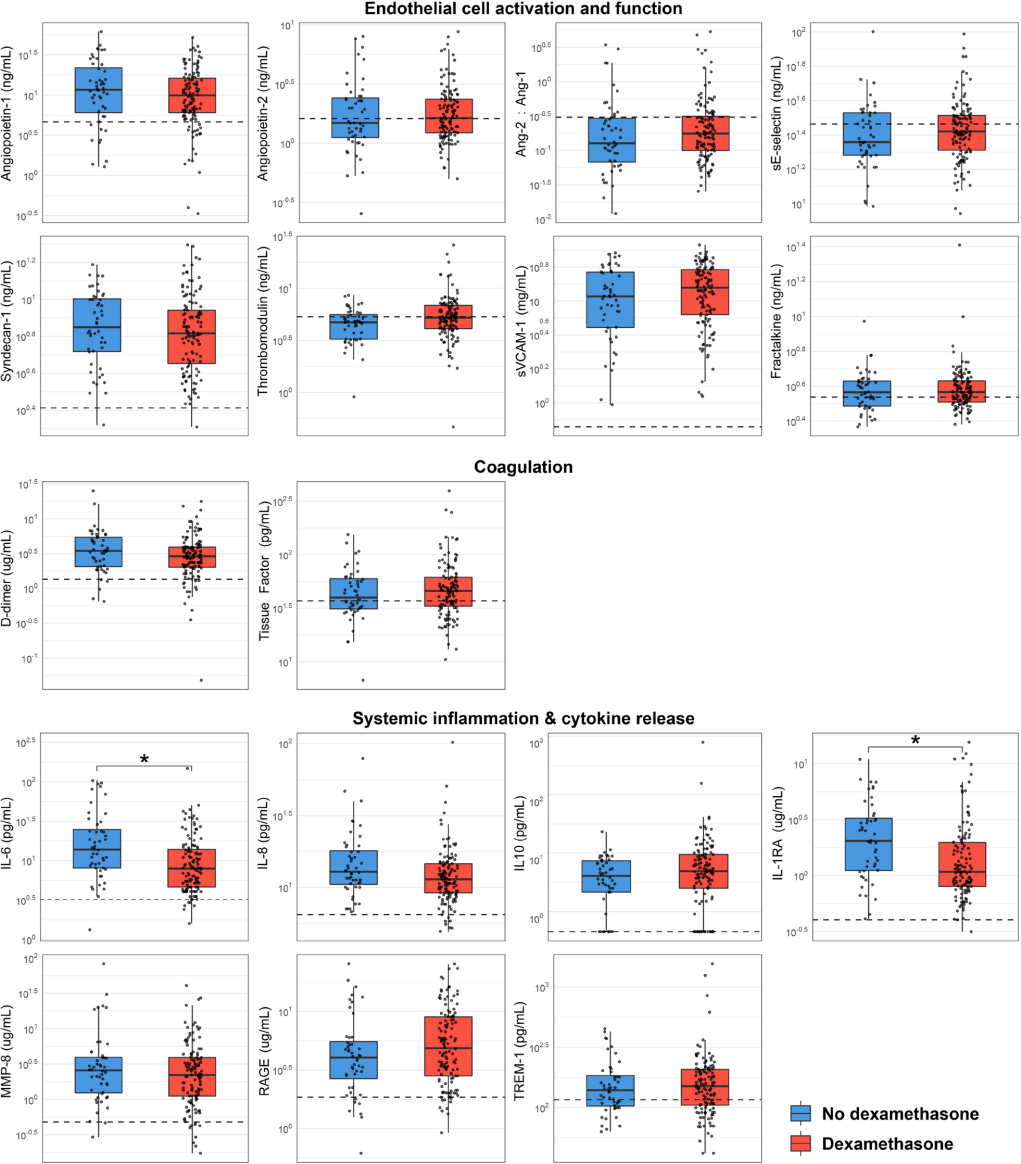
Biomarkers in hospitalized COVID-19 ward patients stratified according to treatment with dexamethasone prior to sampling. Data are expressed as box-whisker diagrams representing the median and 1.5 IQR as whiskers, and individual data points. Dotted lines indicate median values obtained in 21 age-matched outpatient clinic non-infectious controls. * Indicates BH-corrected P < 0.05. IL: interleukin, IL-1RA: interleukin-1 receptor antagonist, MMP-8: matrix metalloproteinase-8, RAGE: receptor for advanced glycation end-products, TREM-1: triggering receptor expressed on myeloid cells-1, VCAM-1: vascular cell adhesion molecule-1

Our primary objective was to compare host response biomarker levels in plasma obtained within 48 h of admission, i.e., after initiation of dexamethasone treatment in wave 2. Biomarker levels reflecting endothelial cell activation and function or activation of the coagulation system did not differ between patients with or without dexamethasone treatment (Fig. 1). However, patients treated with dexamethasone had lower plasma interleukin (IL)-6 concentrations (median [IQR] 7.92 [4.63–13.89] vs 13.81 [8.06–25.01] pg/mL in untreated patients, p = 0.01) and lower plasma IL-1 receptor antagonist concentrations (1.08 [0.80–1.97] vs 2.04 [1.11–3.26] ng/mL, p < 0.01). After correction for possible confounding, dexamethasone treatment was a significant predictor of not only plasma IL-6 and IL-1 receptor antagonist concentrations, but also of IL-8. Other markers of systemic inflammation or cytokines were not significantly different between waves. In a secondary analysis, we analyzed biomarkers in a subset of patients [13 (27%) from wave 1 and 40 (32%) from wave 2] from whom follow-up samples were obtained 3–4 days after admission. At day 3–4, plasma IL-6 and IL-1 receptor antagonist concentrations were not different between groups, whilst thrombomodulin (endothelial injury marker) and soluble TREM-1 (inflammation marker) were higher in dexamethasone-treated patients (Additional file 1: Table).

## Discussion

We here compared the plasma concentrations of 16 host response biomarkers providing insight into dysregulation of key pathways implicated in the pathogenesis of COVID-19 in dexamethasone-treated and untreated patients. Unlike hydrocortisone in sepsis [4], dexamethasone treatment was not associated with attenuated endothelial responses in COVID-19, and dexamethasone did not impact coagulation activation. Dexamethasone treatment was associated with lower plasma IL-6 and IL-1 receptor antagonist levels early after initiation.

A recent observational study in 20 dexamethasone-treated and 12 dexamethasone-untreated COVID-19 patients reported a decrease in angiopoietin-2 and receptor for advanced glycation end-products after dexamethasone treatment [6], biomarkers that were not different between treatment groups in our study. However, unlike our study, this previous investigation involved critically ill patients, had a small sample size and had major baseline differences.

Remarkably, the plasma concentrations of thrombomodulin and TREM-1 were higher at day 3–4 in patients treated with dexamethasone. A clear explanation for this unexpected finding is not available. Dexamethasone did not affect the plasma levels of thrombomodulin in healthy subjects [7], and decreased TREM-1 expression and release from pro-monocytic cells [8].

Our study has strengths and limitations. Data on the effects of dexamethasone on the immune response to SARS-CoV-2 infection are scarce. Whilst patient groups were largely comparable, results are not from a controlled clinical trial. Nevertheless, selection or indication bias is estimated to be minimal due to dexamethasone treatment becoming standard of care from a specific moment in time. The alpha variant (B.1.1.7) became dominant once we terminated enrolling patients in March 2021, minimizing the influence of different SARS-CoV-2 variants. Additionally, correction for demographics, length of symptoms, comorbidity, and diseases severity did not change the results. We compared systemic host responses; analyses of pulmonary responses are not feasible in patients who are not intubated. Our primary analysis focused on host response parameters shortly after hospital admission; nonetheless, measurements in a subgroup 3–4 days after admission did not disclose a mechanism of protective action of dexamethasone either. Notably, in community-acquired pneumonia, dexamethasone influenced plasma cytokine levels early after treatment initiation [9] while in a controlled human inflammation model a single prednisolone dose acutely inhibited cytokine release and endothelial cell activation [10].

Our data argue against modification of vascular-procoagulant responses as an early mechanism of action of dexamethasone in COVID-19. The results of this study could be a rationale for further exploration of the mechanism of action of dexamethasone in COVID-19 in future research.

## Supplementary Information

**Additional file 1: Table S1**. Host response biomarkers at day 3-4 of hospital admission. Results are presented as median [interquartile range]. *IL* interleukin, *IL-1RA* interleukin-1 receptor antagonist, *MMP-8* matrix metalloproteinase-8, *RAGE* receptor for advanced glycation end-products, *TREM-1* triggering receptor expressed on myeloid cells-1, *VCAM-1* vascular cell adhesion molecule-1. * Reasons for missingness were discharged (41.3%), transfer to another hospital (5.8%), initiation of trial medication (10.8%) and no sampling (39.7%).

## Data Availability

The datasets used and/or analyzed during the current study are available from the corresponding author on reasonable request.

## Abbreviations

BH: Benjamini-Hochberg
COVID-19: Coronavirus disease 2019
ICU: Intensive care unit
IL: Interleukin
IL-1RA: Interleukin-1 receptor antagonist
IQR: Interquartile range
MEWS: Modified early warning score
MMP-8: Matrix metalloproteinase-8
qSOFA: Quick sequential organ failure assessment
RAGE: Receptor for advanced glycation end-products
TREM-1: Triggering receptor expressed on myeloid cells-1
VCAM-1: Vascular cell adhesion molecule-1
