# Supplementary material for: Association between dexamethasone treatment and the host response in COVID-19 patients admitted to the general ward
Source: Respir Res. 2022 Jun 3;23:145. doi: 10.1186/s12931-022-02060-3 (PMC9163524; doi:10.1186/s12931-022-02060-3)
Supplement: Supplementary file 1 — Additional file 1: Table S1. Host response biomarkers at day 3-4 of hospital admission. Results are presented as median [interquartile range]. IL interleukin, IL-1RA interleukin-1 receptor antagonist, MMP-8 matrix metalloproteinase-8, RAGE receptor for advanced glycation end-products, TREM-1 triggering receptor expressed on myeloid cells-1, VCAM-1 vascular cell adhesion molecule-1. * Reasons for missingness were discharged (41.3%), transfer to another hospital (5.8%), initiation of trial medication (10.8%) and no sampling (39.7%). [file 12931_2022_2060_MOESM1_ESM.docx]

| **Table S1: Host response biomarkers at day 3-4 of hospital admission** | | |  |
| --- | --- | --- | --- |
|  | **Wave 1**  **(no dexamethasone)** | **Wave 2**  **(dexamethasone)** | **p-value** |
|  | n = 13* | n = 40* |  |
| **Endothelial cell activation and function** | |  |  |
| Angiopoietin-1 (ng/mL) | 10.64 [4.43-25.12] | 12.43 [6.77-21.92] | 0.64 |
| Angiopoietin-2 (ng/mL) | 1.28 [1.16-2.42] | 1.31 [1.00-1.74] | 0.94 |
| Angiopoietin-2:1 ratio | 0.18 [0.06-0.52] | 0.10 [0.06-0.18] | 0.82 |
| Soluble E-selectin (ng/mL) | 26.55 [17.53-32.66] | 24.69 [18.78-31.26] | 0.94 |
| Syndecan-1 (ng/mL) | 10.39 [8.07-10.86] | 9.21 [7.42-13.11] | 0.94 |
| Thrombomodulin (ng/mL) | 4.44 [3.54-5.38] | 6.37 [5.39-8.43] | **0.02** |
| Soluble VCAM-1 (µg/mL) | 2.94 [1.69-3.79] | 4.80 [3.09-5.98] | 0.18 |
| Fractalkine (ng/mL) | 3.65 [3.08-4.24] | 3.70 [3.06-4.56] | 0.94 |
| **Coagulation** |  |  |  |
| D-dimer (µg/mL) | 3.47 [2.18-4.62] | 3.16 [2.20-4.53] | 0.94 |
| Soluble tissue factor (pg/mL) | 38.90 [35.60-60.69] | 52.55 [40.73-80.22] | 0.23 |
| **Systemic inflammation & cytokine release** | |  |  |
| IL-6 (pg/mL) | 11.29 [9.23-13.66] | 7.92 [4.84-15.46] | 0.94 |
| IL-8 (pg/mL) | 10.66 [6.47-17.34] | 11.39 [8.51-15.10] | 0.94 |
| IL-10 (pg/mL) | 2.15 [1.07-3.29] | 3.12 [0.45-5.17] | 0.94 |
| IL-1RA (ng/mL) | 1.70 [1.16-3.47] | 1.60 [0.82-3.54] | 0.94 |
| MMP-8 (ng/mL) | 2.45 [1.14-4.39] | 3.03 [1.86-3.99] | 0.94 |
| Soluble RAGE (ng/mL) | 2.18 [1.95-3.71] | 5.72 [2.26-10.07] | 0.18 |
| Soluble TREM-1 (pg/mL) | 124.8 [98.7-175.1] | 192.8 [151.2-251.7] | **0.02** |

Results are presented as median [interquartile range]. Abbreviations: IL: interleukin, IL-1RA: interleukin-1 receptor antagonist, MMP-8: matrix metalloproteinase-8, RAGE: receptor for advanced glycation end-products, TREM-1: triggering receptor expressed on myeloid cells-1, VCAM-1: vascular cell adhesion molecule-1. * Reasons for missingness were discharged (41.3%), transfer to another hospital (5.8%), initiation of trial medication (10.8%) and no sampling (39.7%).
